# Sex-specific associations of the controlling nutritional status score with diabetic kidney disease among Chinese individuals: a retrospective cross-sectional study

**DOI:** 10.3389/fnut.2025.1662140

**Published:** 2025-09-05

**Authors:** Yu-Nan Han, Tong Wang, Qin Lin, Lin Li, Yan-Rui Ren

**Affiliations:** ^1^Department of Endocrinology, The First Affiliated Hospital of Yangtze University, Jingzhou, Hubei, China; ^2^Department of Medicine, Yangtze University, Jingzhou, Hubei, China

**Keywords:** sex-specific differences, CONUT, DKD, nutritional risk assessment, T2DM

## Abstract

**Background:**

Recognizing the crucial role of nutritional status in the advancement of diabetic complications, this investigation aimed to evaluate sex-specific disparities concerning the relationship between the Controlling Nutritional Status (CONUT) score and diabetic kidney disease (DKD).

**Methods:**

Data obtained from the First Affiliated Hospital of Yangtze University, spanning January 2022 to May 2024, were utilized. The CONUT score was determined utilizing serum albumin (ALB), total cholesterol (TC), and lymphocyte (LYM) count. Sex-specific correlations among CONUT scores and DKD were evaluated using multivariate logistic regression and restricted cubic splines (RCS). Receiver operating characteristic (ROC) curve analysis was employed to ascertain the area under the curve (AUC) for the CONUT score in female participants. Subgroup analyses and interaction assessments were conducted to investigate the influence of the CONUT score within sex-specific subgroups.

**Results:**

A total of 1,429 individuals were enrolled. Following adjustment for all covariates, women within the elevated CONUT score group exhibited a 66% augmented odds of DKD [OR = 1.66 (95%CI: 1.08–2.58)]. RCS analysis indicated a linear positive correlation between the CONUT score and the odds of DKD in women (P-non-linear: 0.840). The AUC for the CONUT score in women was 0.700 (95%CI: 0.653–0.748), indicating its potential utility as a risk identification tool for DKD. Subgroup analyses revealed a noteworthy positive correlation between elevated CONUT scores and the odds of DKD among females aged ≥60 years, those with a high school diploma, who smoked, consumed alcohol, were not hypertensive, had hyperlipidemia, or had a high TC/HDL ratio.

**Conclusion:**

This study demonstrates sex-specific disparities in the prevalence of DKD associated with elevated CONUT scores. These outcomes underscore the significance of individualized nutritional interventions for females at an elevated odd of DKD.

## 1 Introduction

Type 2 diabetes mellitus (T2DM) is defined as a chronic metabolic disorder primarily characterized by sustained hyperglycemia (1). Globally, the prevalence of T2DM is rapidly escalating, presenting a substantial public health concern (2). As of 2021, a~140.9 million adults in China were estimated to be living with the condition, with projections indicating an increase to ~174.4 million individuals by 2045 (3). Long-term hyperglycemia in individuals with T2DM can lead to the development of various severe complications, including nephropathy, cardiovascular disease, neuropathy, and retinopathy (4, 5). Among these, diabetic kidney disease (DKD) is identified as a significant microvascular complication frequently observed in T2DM, and it has become a leading etiology for both chronic kidney disease (CKD) and end-stage renal disease (ESRD) (6–9). This complication is observed in ~40% of individuals with T2DM.

The nutritional status of patients with T2DM is frequently compromised due to diabetes-related complications and comorbidities (10). An 11.1% higher incidence of malnutrition has been reported in CKD patients with concurrent diabetes compared to those without diabetes (11). Evidence suggests that nutritional therapy can delay the appearance of early CKD symptoms and disease progression, and, in advanced stages, the need for renal replacement therapy may be postponed (12). Consequently, the maintenance and improvement of nutritional status are considered crucial for the management of CKD in diabetic patients (13). The Controlling Nutritional Status (CONUT) score is a widely utilized immunonutritional marker. It facilitates a comprehensive assessment of an individual's chronic inflammatory status, immune function, and nutritional status through the combined evaluation of total cholesterol (TC), serum albumin (ALB), and lymphocyte count (LYM) (14). Previous reports have indicated that the CONUT score is a reliable prognostic predictor and has demonstrated superior performance compared to the prognostic nutritional index (PNI) in several malignancies (15, 16). Notably, the CONUT score has garnered increasing recognition as a valuable indicator in metabolic health. Studies have shown that even among individuals with overweight or obesity, the CONUT score can identify underlying malnutrition, which is independently associated with adverse outcomes, demonstrating its capacity to provide critical prognostic information beyond traditional anthropometric measures such as BMI (17). Furthermore, the CONUT score has been found to be significantly elevated in T2DM patients compared to healthy individuals, and importantly, it serves as an independent indicator for the presence of diabetic microvascular complications, demonstrating strong diagnostic utility for these complications (18). These findings collectively underscore the importance of CONUT in reflecting comprehensive nutritional status and its critical role in assessing the risk of diabetic complications.

It is notable that sex-associated variations are instrumental in the development and advancement of DKD. Current investigations into the relationship between DKD and biological sex have produced disparate findings. Generally, differences in DKD risk and disease progression may be observed between male and female individuals; however, specific conclusions are frequently contingent upon various factors, including the particular type of diabetes, age, and hormonal status. For instance, certain research has indicated that male patients with type 1 diabetes mellitus (T1DM) exhibit a greater propensity for progression to albuminuric DKD (19). Concurrently, other research suggests that women may face an elevated risk of DKD, even when diabetes is well-controlled (20). Regarding individuals with T2DM, the impact of sex differences is more intricate. Evidence has shown that women may exhibit a more rapid rate of DKD progression after menopause, which could be attributed to decreased estrogen levels and other physiological changes (21). Furthermore, variations in metabolism and immune responses between sexes may also influence the interplay between nutritional status and DKD. Therefore, exploring sex differences in the impact of the CONUT score on DKD in patients with T2DM is considered to have significant clinical implications as it could facilitate the development of more targeted nutritional intervention strategies.

The association between the CONUT score and DKD was investigated in this study, employing a retrospective analysis of data from Chinese patients diagnosed with T2DM. This research is intended to address the existing scarcity of data concerning the Chinese population and will place particular emphasis on the analysis of sex differences, with the ultimate goal of providing more precise guidance for clinical practice.

## 2 Materials and methods

### 2.1 Ethics statement

Ethics approval for this investigation was procured from the Ethics Committee of the First Affiliated Hospital of Yangtze University (KY2025-039-01). The research adhered to both institutional and national ethical guidelines for research, in alignment with the stipulations of the 1964 Declaration of Helsinki and its subsequent amendments, or equivalent ethical frameworks. As this study constituted a retrospective analysis of clinical data, the requirement for written informed consent was waived.

### 2.2 Data source and participants

Retrospective data involving 1,429 T2DM individuals admitted to the Department of Endocrinology and Clinical Nutrition at the First Affiliated Hospital of Yangtze University between January 2022 and May 2024 were analyzed. This patient group consisted of 866 males and 563 females. Each participant fulfilled the 1999 World Health Organization (WHO) diagnostic criteria for T2DM (22).

These criteria include the following: (1) presentation of typical diabetes symptoms with a random plasma glucose level ≥11.1 mmol/L; (2) a fasting plasma glucose level ≥7.0 mmol/L; or (3) a plasma glucose level ≥11.1 mmol/L 2 h after an oral glucose tolerance test or the ingestion of 75 g of glucose. For patients without any typical diabetes symptoms, repeat testing was required on another day. According to the Chinese guidelines for the prevention and treatment of DKD (23), a diagnosis of DKD was established when renal impairment was primarily attributed to diabetes, with the exclusion of other CKD etiologies. In addition, at least one of the subsequent criteria had to be fulfilled: (1) a urinary albumin-to-creatinine ratio (UACR) of ≥30 mg/g observed in a minimum of two out of three measurements, following the careful elimination of any confounding variables; (2) an estimated glomerular filtration rate (eGFR) below 60 mL/min^−1^ (1.73m^2^)^−1^, sustained for a period exceeding 3 months; or (3) renal biopsy results demonstrating characteristic DKD histopathological alterations.

Participants were excluded from this study if they presented with any of the following: (1) T1DM, gestational diabetes, or other specified forms of diabetes; (2) acute diabetic complications, such as diabetic ketoacidosis, hyperosmolar hyperglycemic state (HHS), lactic acidosis, or hypoglycemic coma; (3) urinary tract infections, hematuria (including during menstruation), or CKD/ESRD not attributable to DKD that necessitated dialysis; (4) hematological disorders or malignant neoplasms; or (5) a hospital stay of <1 day. All individuals meeting any of these exclusion criteria were systematically removed from the dataset during the data collection phase.

### 2.3 Collection of demographic, clinical, and biometric data

Detailed demographic and medical history data were compiled, including sex, patient age, height, weight, educational attainment (categorized as <high school diploma, high school diploma/equivalent, or >high school diploma), marital status (e.g., unmarried, married/cohabiting, and widowed/divorced/separated), smoking history (yes/no), prior alcohol intake (yes/no), systolic blood pressure (SBP), diastolic blood pressure (DBP), specific diabetes-related complications (retinopathy, peripheral neuropathy, and diabetic foot), hypertension (yes/no), dyslipidemia (yes/no), cardiovascular disease (yes/no), and medication history (no medication, insulin only, oral medication and insulin, oral medication only). For the purpose of biochemical assessment, all participants were instructed to fast for a minimum of 8 h, followed by venous blood sampling on the subsequent morning. The biochemical parameters subsequently determined encompassed white blood cell count (WBC), neutrophil count (NEU), LYM, serum ALB, total bilirubin (TB), urea (UREA), creatinine (CR), uric acid (UA), alanine aminotransferase (ALT), aspartate aminotransferase (AST), gamma-glutamyl transferase (GGT), microalbumin (μALB), UACR, glycated hemoglobin (HbA1c), fasting blood glucose (FBG), postprandial blood glucose (PBG), fasting insulin (FI), 2-h postprandial C-peptide (2h-CP), total fat (TF), fasting C-peptide (FCP), thyroid-stimulating hormone (TSH), free triiodothyronine (FT3), free thyroxine (FT4), high-density lipoprotein (HDL), low-density lipoprotein (LDL), triglycerides (TG), and TC.

To ensure the accuracy and reliability of biochemical measurements, the following instruments and methods were employed: WBC, NEU, LYM, and ALB levels were determined using a BAYER five-part differential hematology analyzer and a CELL-DYN 1700 three-part differential hematology analyzer. TB, UREA, CR, UA, ALT, AST, GGT, μALB, HbA1c, FBG, PBG, TF, HDL, LDL, TG, and TC levels were measured using an OLYMPUS AU600 automated biochemical analyzer and a Roche Diagnostics MODULAR PP automated biochemical analyzer (D module). UACR was assessed using an OLYMPUS AU600 automated biochemical analyzer, a Roche Diagnostics MODULAR PP automated biochemical analyzer (D module), and a UF-50 automated urine formed element analyzer/urine sediment analyzer. FI, 2h-CP, FCP, TSH, FT3, and FT4 levels were detected using an ABBOTT AXSYM automated immunoassay analyzer.

### 2.4 Assessment of CONUT

For this retrospective cross-sectional study, the Controlling Nutritional Status (CONUT) score was chosen as our primary nutritional assessment tool due to its objective nature and reliance on routinely collected laboratory parameters. This approach ensured high data availability and feasibility for analysis within our existing electronic medical records, thereby minimizing the challenges associated with missing subjective or anthropometric data that more comprehensive tools (e.g., Nutritional Risk Screening 2002 [NRS-2002] and Mini Nutritional Assessment [MNA]) would typically require.

The CONUT score is determined based on three parameters: serum ALB, TC, and LYM count. An optimal cutoff of 3.5 for the CONUT score was determined through receiver operating characteristic (ROC) curve analysis performed on our study population. This approach aligns with the established methodology for assessing the CONUT score's utility and determining optimal thresholds in various clinical contexts (24, 25). Accordingly, participants were stratified into two groups: those with a CONUT score <3.5 and those with a score of ≥3.5. Comprehensive information pertaining to the CONUT score is detailed in Supplementary Table S1.

### 2.5 Assessment of prognostic nutritional index (PNI)

In parallel to the CONUT score, the Prognostic Nutritional Index (PNI), another widely recognized immunonutritional assessment tool, was calculated for each participant. The PNI was computed using the following formula: Albumin (g/dL) + 5 × Total lymphocyte count (10^9^/L). To ensure methodological consistency with the CONUT score, an optimal cutoff for PNI was determined through ROC curve analysis in the entire study population, aiming to categorize participants into two groups (PNI <50.55 and PNI ≥ 50.55) for subsequent statistical analyses.

### 2.6 Statistical analysis

All statistical computations were carried out using R version 4.4.1 software (R Foundation for Statistical Computing, Vienna, Austria). Quantitative data are reported as medians (P25, P75), while qualitative variables are presented as counts (percentages). Comparisons of categorical data between groups were conducted utilizing Pearson's chi-squared test. Considering that the continuous variables subjected to analysis exhibited a non-normal distribution, as confirmed by the Anderson-Darling test, the Mann–Whitney U-test was employed to evaluate inter-group differences.

The relationship between the CONUT score and the propensity for DKD within the overall study population was assessed via multivariate logistic regression models, with findings reported as odds ratios (ORs) and their respective 95% confidence intervals (CIs). Furthermore, analyses stratified by sex were executed. For the purposes of this investigation, the CONUT score was bifurcated into two categories for analysis: <3.5 and ≥3.5. Hierarchical regression models were developed through sequential adjustments for various covariates: Model 1 represented the unadjusted analysis; Model 2 incorporated adjustments for sex, age, educational attainment, and marital status; and building upon Model 2, Model 3 further accounted for BMI, hypertension, dyslipidemia, cardiovascular disease (CVD), HbA1c, diabetic retinopathy (DR), diabetic peripheral neuropathy (DPN), total fat (TF), UACR, UA, medication status, smoking habits, and alcohol consumption. The presence of multicollinearity across all constructed models was evaluated using the variance inflation factor (VIF) and generalized variance inflation factor (GVIF). As detailed in Supplementary Tables S2, S3, the results indicated that all VIF values (for variables with a single degree of freedom) and adjusted GVIF [GVIF^∧^(1/(2*Df*))] values (for categorical variables with multiple degrees of freedom) for all independent variables were well below the commonly accepted thresholds [e.g., VIF <10 and GVIF^∧^(1/(2*Df*)) <2], suggesting the absence of significant multicollinearity. Furthermore, restricted cubic spline (RCS) models were employed both in the overall study population and within sex-specific strata to evaluate the non-linear relationship between the CONUT score and DKD. In parallel, the association between the PNI and DKD was analyzed using the same multivariate logistic regression models and sex-specific stratification approaches as employed for the CONUT score. Subsequently, separate subgroup analyses were conducted for female and male participants to assess the potential modifying effects of key demographic and clinical variables on the association between the CONUT score and the outcome. All covariates, excluding those specifically utilized for stratification, were adjusted for in the statistical models. The aforementioned analyses incorporated stratification by age (<60 or ≥60 years), educational attainment (categorized as <high school diploma, high school diploma/equivalent, or >high school diploma), BMI (<25 or ≥25 kg/m^2^), smoking status (yes/no), alcohol intake (yes/no), hypertension (yes/no), dyslipidemia (yes/no), and CVD (yes/no). In addition, to address the potential influence of fat distribution and metabolic health, we performed subgroup analyses stratified by the triglyceride-to-HDL cholesterol ratio (TG/HDL ratio) and the total cholesterol-to-HDL cholesterol ratio (TC/HDL ratio). For these ratios, participants were dichotomized based on the median value within their respective sex-specific subgroups. The reciprocal influence between the CONUT score and these variables was evaluated by integrating specific multiplicative terms within the multivariate logistic regression framework. More precisely, multiplicative interaction terms (CONUT score × covariate) were integrated to ascertain if the association between the CONUT score and DKD was modulated by these other factors. The statistical significance of these interactions was determined using analysis of variance. To comprehensively evaluate and compare the prognostic ability of both CONUT and PNI for DKD, ROC curve analysis was performed across the entire study population and within sex-specific subgroups for each score. The area under the ROC curve (AUROC) and its 95% confidence interval (CI) were computed. The statistical significance of differences between the AUCs of CONUT and PNI was assessed using DeLong's test. Finally, propensity score matching (PSM) was utilized to mitigate potential selection bias and confounding factors. Propensity scores were derived from a logistic regression model encompassing pertinent covariates. Subsequently, participants were paired at a 1:2 ratio using a nearest-neighbor matching algorithm, based on these calculated propensity scores. Balance after matching was assessed by comparing the standardized mean differences (SMD) of covariates between the matched groups, with an SMD <0.1 indicating acceptable balance. Subsequently, the matched data underwent analysis using logistic regression models to evaluate the relationship between the CONUT score and DKD across both sexes. This approach aimed to diminish potential biases and bolster the robustness of the study's conclusions. Statistical significance was defined as a two-sided *P*-value <0.05.

## 3 Results

### 3.1 Baseline characteristics of participants

Table 1 provides an overview of the baseline features of the study participants. Among the 1,429 individuals, 60.6% were male, 52.97% were under 60 years of age, 43.53% had attained a high school diploma or higher educational qualification, and 61.58% were married. Significant variations (*P* < 0.05) were noted between the two groups concerning age, education, DR, dyslipidemia, CVD, alcohol consumption, medication use, height, weight, DBP, WBC, NEU, LYM, ALB, UREA, ALT, GGT, UACR, FBG, 2h-CP, TSH, FT3, HDL, TG, and TC.

**Table 1:** Baseline information from the DKD and non-DKD groups.

**Variables**	**Total (*n* = 1,429)**	**Non-DKD (*n* = 490)**	**DKD (*n* = 939)**	**Statistic**	***P***
**Gender**, ***n*** **(%)**				*χ^2^* = 3.35	0.067
Female	563 (39.40)	177 (36.12)	386 (41.11)		
Male	866 (60.60)	313 (63.88)	553 (58.89)		
**Age**, ***n*** **(%)**				χ^2^ = 36.93	<0.001
<60	757 (52.97)	314 (64.08)	443 (47.18)		
≥60	672 (47.03)	176 (35.92)	496 (52.82)		
**Education**, ***n*** **(%)**				*χ^2^* = 10.73	0.005
<High school diploma	338 (23.65)	94 (19.18)	244 (25.99)		
>High school diploma	622 (43.53)	238 (48.57)	384 (40.89)		
High school diploma/equivalent	469 (32.82)	158 (32.24)	311 (33.12)		
**Marital**, ***n*** **(%)**				*χ^2^* = 3.45	0.178
Married/cohabitation	880 (61.58)	317 (64.69)	563 (59.96)		
Unmarried	81 (5.67)	28 (5.71)	53 (5.64)		
Widow/divorce/separation	468 (32.75)	145 (29.59)	323 (34.40)		
**DR**, ***n*** **(%)**				*χ^2^* = 41.25	<0.001
No	893 (62.49)	362 (73.88)	531 (56.55)		
Yes	536 (37.51)	128 (26.12)	408 (43.45)		
**DPN**, ***n*** **(%)**				*χ^2^* = 0.74	0.388
No	888 (62.14)	312 (63.67)	576 (61.34)		
Yes	541 (37.86)	178 (36.33)	363 (38.66)		
**DF**, ***n*** **(%)**				*χ^2^* = 2.56	0.109
No	1,396 (97.69)	483 (98.57)	913 (97.23)		
Yes	33 (2.31)	7 (1.43)	26 (2.77)		
**Hypertension**, ***n*** **(%)**				*χ^2^* = 2.43	0.119
No	668 (46.75)	243 (49.59)	425 (45.26)		
Yes	761 (53.25)	247 (50.41)	514 (54.74)		
**Hyperlipidemia**, ***n*** **(%)**				χ^2^ = 7.55	0.006
No	581 (40.66)	175 (35.71)	406 (43.24)		
Yes	848 (59.34)	315 (64.29)	533 (56.76)		
**CVD**, ***n*** **(%)**				*χ^2^* = 23.42	<0.001
No	587 (41.08)	244 (49.80)	343 (36.53)		
Yes	842 (58.92)	246 (50.20)	596 (63.47)		
**Smoke**, ***n*** **(%)**				*χ^2^* = 1.51	0.219
No	662 (46.33)	216 (44.08)	446 (47.50)		
Yes	767 (53.67)	274 (55.92)	493 (52.50)		
**Drink**, ***n*** **(%)**				*χ^2^* = 4.57	0.033
No	715 (50.03)	226 (46.12)	489 (52.08)		
Yes	714 (49.97)	264 (53.88)	450 (47.92)		
**Medication status**, ***n*** **(%)**				*χ^2^* = 46.27	<0.001
Insulin only	35 (2.45)	13 (2.65)	22 (2.34)		
No medication	196 (13.72)	103 (21.02)	93 (9.90)		
Oral medication and insulin	677 (47.38)	183 (37.35)	494 (52.61)		
Oral medication only	521 (36.46)	191 (38.98)	330 (35.14)		
BMI, M (Q_1_, Q_3_)	24.65 (22.72, 27.01)	24.80 (22.86, 27.28)	24.57 (22.62, 26.75)	Z = −1.56	0.119
CM, M (Q_1_, Q_3_)	1.65 (1.59, 1.70)	1.66 (1.60, 1.71)	1.65 (1.59, 1.70)	Z = −2.22	0.026
KG, M (Q_1_, Q_3_)	67.00 (60.00, 75.00)	68.00 (60.00, 77.00)	67.00 (60.00, 75.00)	Z = −2.43	0.015
DBP, M (Q_1_, Q_3_)	81.00 (74.00, 89.00)	82.00 (76.00, 90.00)	81.00 (73.00, 88.00)	Z = −3.51	<0.001
SBP, M (Q_1_, Q_3_)	132.00 (121.00, 146.00)	132.00 (119.00, 145.00)	133.00 (121.00, 146.00)	Z = −1.21	0.224
WBC, M (Q_1_, Q_3_)	5.78 (4.88, 6.95)	5.99 (5.05, 7.20)	5.67 (4.79, 6.75)	Z = −3.73	<0.001
NEU, M (Q_1_, Q_3_)	3.36 (2.66, 4.22)	3.45 (2.71, 4.27)	3.31 (2.65, 4.18)	Z = −2.26	0.024
LYM, M (Q_1_, Q_3_)	1.74 (1.38, 2.12)	1.83 (1.44, 2.23)	1.70 (1.34, 2.05)	Z = −4.37	<0.001
ALB, M (Q_1_, Q_3_)	4.10 (3.89, 4.34)	4.16 (3.94, 4.37)	4.08 (3.87, 4.33)	Z = −3.87	<0.001
TB, M (Q_1_, Q_3_)	12.90 (10.00, 16.40)	13.00 (10.20, 16.70)	12.90 (9.90, 16.25)	Z = −0.93	0.353
UREA, M (Q_1_, Q_3_)	5.90 (4.86, 7.15)	5.70 (4.75, 6.83)	5.96 (4.90, 7.32)	Z = −2.60	0.009
CR, M (Q_1_, Q_3_)	68.70 (56.00, 82.70)	67.20 (56.30, 80.88)	69.40 (55.85, 84.15)	Z = −1.63	0.102
UA, M (Q_1_, Q_3_)	311.70 (258.90, 376.50)	316.60 (266.88, 382.28)	308.40 (254.00, 374.35)	Z = −1.62	0.106
ALT, M (Q_1_, Q_3_)	14.00 (9.00, 22.00)	14.00 (10.00, 23.00)	14.00 (9.00, 21.00)	Z = −2.15	0.032
AST, M (Q_1_, Q_3_)	18.00 (15.00, 22.00)	17.00 (14.00, 22.00)	18.00 (15.00, 22.00)	Z = −0.55	0.585
GGT, M (Q_1_, Q_3_)	23.00 (16.00, 35.00)	26.00 (17.00, 40.00)	21.00 (15.00, 33.00)	Z = −4.74	<0.001
μALB, M (Q_1_, Q_3_)	8.93 (5.23, 31.40)	8.72 (5.25, 24.96)	9.14 (5.23, 33.52)	Z = −0.95	0.341
UACR, M (Q_1_, Q_3_)	9.23 (5.23, 30.87)	7.74 (4.83, 20.49)	10.12 (5.60, 39.31)	Z = −4.27	<.001
HbA1c, M (Q_1_, Q_3_)	8.30 (7.10, 9.90)	8.30 (7.10, 9.90)	8.30 (7.10, 9.90)	Z = −0.27	0.784
FBG, M (Q_1_, Q_3_)	8.00 (6.55, 9.95)	7.88 (6.48, 9.52)	8.06 (6.61, 10.15)	Z = −2.21	0.027
PBG, M (Q_1_, Q_3_)	13.94 (11.17, 17.27)	13.75 (10.99, 16.84)	13.98 (11.21, 17.49)	Z = −1.56	0.119
FI, M (Q_1_, Q_3_)	7.69 (4.88, 12.48)	7.60 (4.91, 11.75)	7.75 (4.85, 12.79)	Z = −0.57	0.566
2h-CP, M (Q_1_, Q_3_)	3.24 (2.19, 4.50)	3.40 (2.31, 4.62)	3.15 (2.11, 4.40)	Z = −2.56	0.01
TF, M (Q_1_, Q_3_)	62.17 (42.13, 92.97)	64.30 (42.37, 98.16)	59.69 (42.09, 90.60)	Z = −1.51	0.131
FCP, M (Q_1_, Q_3_)	1.58 (1.10, 2.14)	1.60 (1.15, 2.20)	1.57 (1.10, 2.12)	Z = −1.06	0.291
TSH, M (Q_1_, Q_3_)	1.75 (1.20, 2.60)	1.71 (1.18, 2.45)	1.78 (1.21, 2.66)	Z = −2.01	0.044
FT3, M (Q_1_, Q_3_)	2.65 (2.42, 2.87)	2.70 (2.46, 2.93)	2.62 (2.40, 2.84)	Z = −3.51	<0.001
FT4, M (Q_1_, Q_3_)	13.69 (12.67, 14.73)	13.76 (12.75, 14.91)	13.66 (12.62, 14.63)	Z = −1.58	0.114
HDL, M (Q_1_, Q_3_)	1.07 (0.91, 1.28)	1.04 (0.88, 1.22)	1.08 (0.92, 1.29)	Z = −3.41	<0.001
LDL, M (Q_1_, Q_3_)	2.32 (1.75, 2.82)	2.36 (1.83, 2.83)	2.30 (1.73, 2.82)	Z = −1.69	0.092
TG, M (Q_1_, Q_3_)	1.65 (1.16, 2.40)	1.72 (1.23, 2.61)	1.58 (1.12, 2.25)	Z = −3.23	0.001
TC, M (Q_1_, Q_3_)	80.17 (67.20, 93.50)	82.33 (69.59, 94.22)	79.45 (65.76, 92.96)	Z = −2.51	0.012

Z: Mann–Whitney test, χ^2^: chi-square test.

M: median, Q_1_: 1st quartile, Q_3_: 3rd quartile.

BMI, body mass index; DBP, diastolic blood pressure; SBP, systolic blood pressure; WBC, white blood cell; NEU, neutrophil; LYM, lymphocyte; ALB, albumin; TB, total bilirubin; Cr, creatinine; UA, uric acid; ALT, alanine aminotransferase; AST, aspartate aminotransferase; GGT, gamma-glutamyl transferase; μALB, microalbumin; UACR, urinary albumin-to-creatinine ratio; FBG, fasting blood glucose; PBG, postprandial blood glucose; FI, fasting insulin; 2h-CP, postprandial C-peptide; TF, total fat; FCP, fasting C-peptide; TSH, thyroid-stimulating hormone; FT3, free triiodothyronine; FT4, free thyroxine; HDL, high-density lipoprotein; LDL, low-density lipoprotein; TG, triglyceride; TC, total cholesterol.

### 3.2 Association of CONUT score with diabetic kidney disease

The link between the CONUT score and DKD was evaluated employing multivariate logistic regression models, with the results detailed in Table 2. It was observed that a CONUT score ≥3.5 was significantly correlated with an elevated likelihood of DKD relative to a CONUT score <3.5 in Model 1 (OR = 1.50, 95%CI = 1.19–1.89) and Model 2 (OR = 1.30, 95%CI = 1.02–1.66). However, in the fully adjusted model, no significant relationship was detected between the CONUT score and DKD (OR = 1.21, 95% CI = 0.94–1.57).

**Table 2:** Association between CONUT score and diabetic kidney disease (DKD).

**Variable**	**Characteristic**	**Model 1 OR (95%CI)**	***P*-value**	**Model 2 OR (95%CI)**	***P*-value**	**Model 3 OR (95%CI)**	***P*-value**
Overall	CONUT						
	<3.5	Ref		Ref		Ref	
	≥3.5	1.50 (1.19, 1.89)	<0.001	1.30 (1.02, 1.66)	0.032	1.21 (0.94, 1.57)	0.14
Female	CONUT						
	<3.5	Ref		Ref		Ref	
	≥3.5	1.60 (1.09, 2.35)	0.017	1.50 (1.02, 2.23)	0.043	1.66 (1.08, 2.58)	0.023
Male	CONUT						
	<3.5	Ref		Ref		Ref	
	≥3.5	1.44 (1.07, 1.94)	0.016	1.19 (0.87, 1.62)	0.3	1.07 (0.77, 1.49)	0.7

Data are presented as weighted odds ratios (OR) with 95% confidence intervals (CI). Model 1 is the crude model. Model 2 is adjusted for gender, age, education, and marital status. Model 3 is further adjusted for BMI, hypertension, hyperlipidemia, CVD, HbA1c, DR, DPN, TF, UACR, UA, medication status, smoking, and alcohol consumption.

### 3.3 Sex-specific association of CONUT with diabetic kidney disease

**Figure 1:**
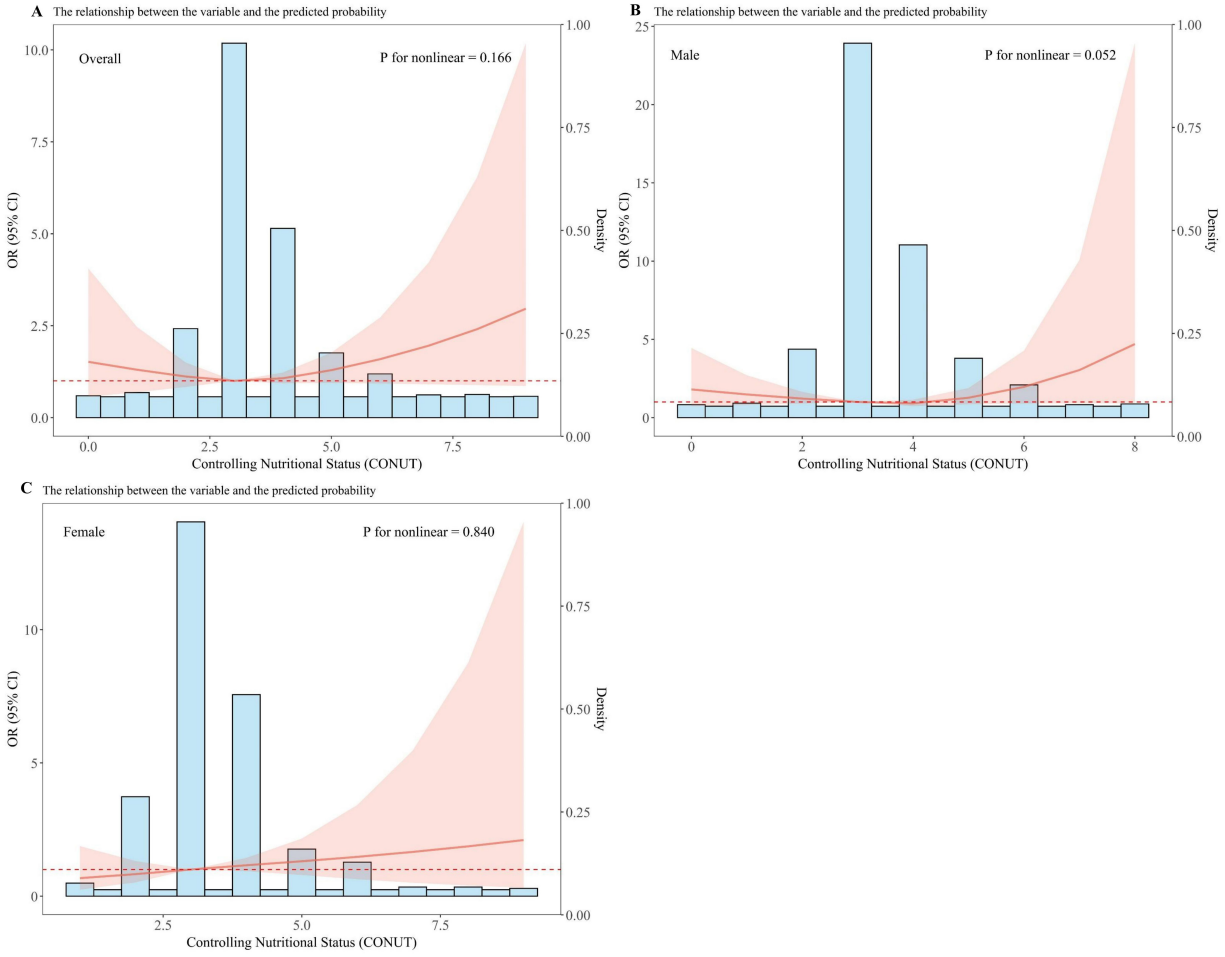
Dose–response relationship between CONUT and overall odds of DKD and sex-specific odds of DKD among Chinese individuals. The model is adjusted for age, education, marital status, BMI, hypertension, hyperlipidemia, CVD, HbA1c, DR, DPN, TF, UACR, UA, medication status, smoking, and alcohol consumption. The central estimates are represented by the solid red line, the red shaded area represents the 95% confidence intervals, and the frequency density is depicted by the blue bar graph. **(A)** Dose–response relationship between CONUT and the total population with DKD. **(B)** Dose–response relationship between CONUT and male patients with DKD. **(C)** Dose–response relationship between CONUT and female patients with DKD.

Sex-specific differences were observed regarding the association of the CONUT score with DKD, as detailed in Table 2. Following progressive covariate adjustment, an elevated CONUT score appeared to confer heightened odds for females relative to males. Specifically, among female participants, those in the higher CONUT stratum (CONUT ≥ 3.5) demonstrated an increased odds of DKD by 60% [OR = 1.60 (95%CI: 1.09–2.35)], 50% [OR = 1.50 (95%CI: 1.02–2.23)], and 66% [OR = 1.66 (95%CI: 1.08–2.58)] across different models, when contrasted with the lower CONUT category (CONUT <3.5). However, no significant association between the CONUT score and DKD was discernible among male participants. Furthermore, multivariate-adjusted RCS plots demonstrated linear patterns regarding the CONUT score's relationship with the odds of DKD across the entire study group and within sex-specific strata (Figure 1). A linear positive association was identified between the CONUT score and DKD occurrence for the overall group (P-non-linear: 0.166), male participants (P-non-linear: 0.052), and female participants (P-non-linear: 0.840).

### 3.4 Sex-specific association of PNI with diabetic kidney disease

The association between the Prognostic Nutritional Index (PNI) and the odds of diabetic kidney disease (DKD) was evaluated using multivariate logistic regression. In the overall study population, a higher PNI score (PNI ≥ 50.55) was significantly associated with a reduced odds of DKD [OR = 0.74 (95%CI: 0.58–0.95), *P* = 0.018]. This indicates that PNI is an independent protective factor against DKD in the total group. However, when stratified by sex, this significant association was not observed. For females, the PNI's association was non-significant [OR = 0.70 (95%CI: 0.46–1.06), *P* = 0.090] and similarly for males [OR = 1.07 (95%CI: 0.77–1.49), *P* = 0.057]. This sex-specific pattern of association for PNI, which showed significance only in the overall population but not in sex-stratified analyses, contrasts with the CONUT score's findings (Supplementary Table S4).

### 3.5 Sex stratified ROC curve analysis

**Figure 2:**
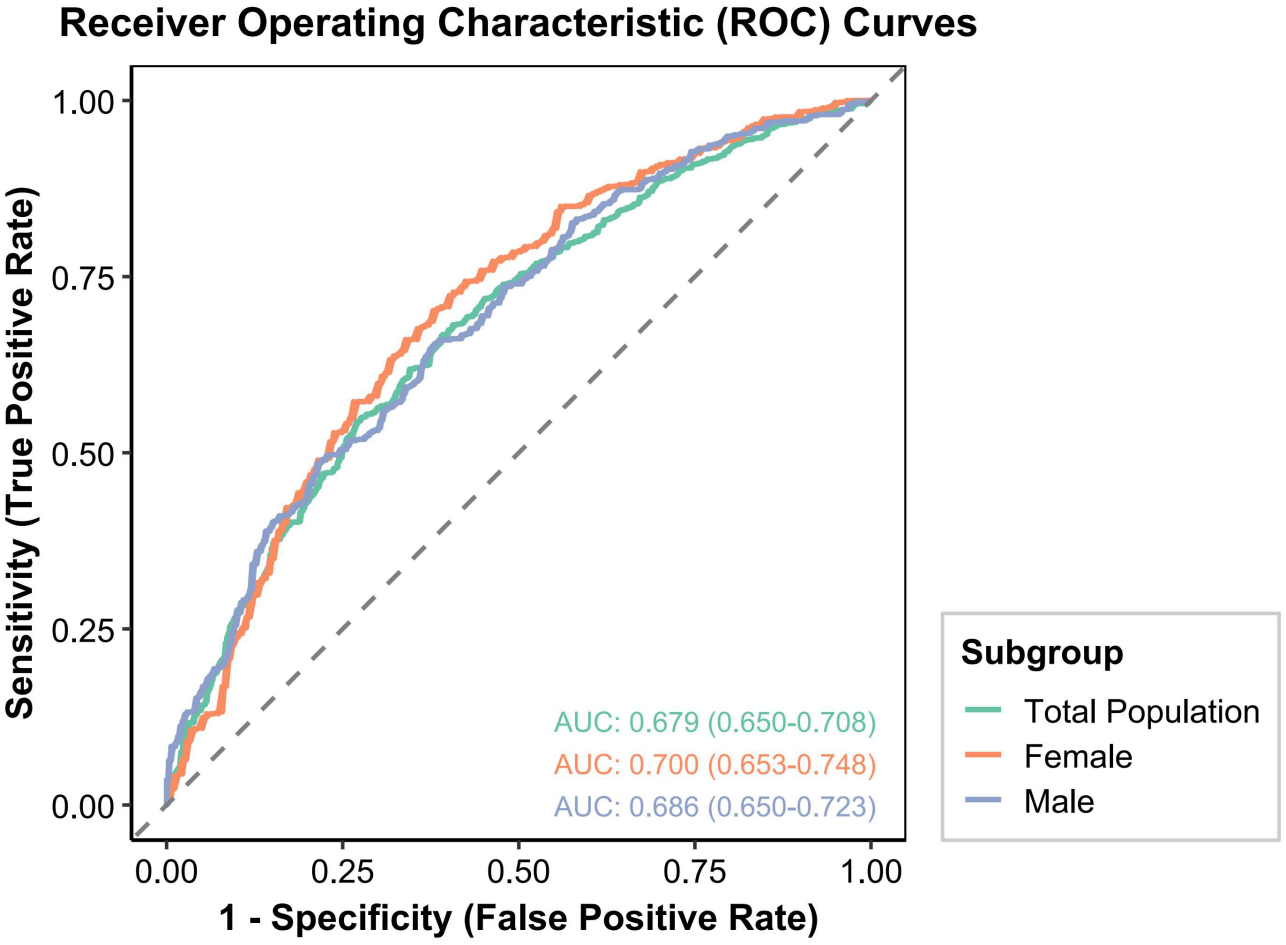
ROC curves for the prediction of DKD by the CONUT score in the overall population, males, and females among Chinese individuals.

The prognostic capability of the CONUT score for DKD was assessed via ROC curve analysis (Figure 2). In the female subgroup, the AUC for the CONUT score was 0.700 (95%CI: 0.653–0.748), suggesting its potential as a risk identification tool for DKD. In the male subgroup, the AUC was 0.686 (95%CI: 0.650–0.723), indicating comparable predictive performance. For the overall population, the AUC was 0.679 (95%CI: 0.650–0.708), demonstrating acceptable overall predictive performance. However, DeLong tests revealed no statistically significant differences in AUCs when comparing the female subgroup with the male subgroup (*p* = 0.640), the female subgroup with the overall population (*p* = 0.460), or the male subgroup with the overall population (*p* = 0.777). While a numerically higher AUC was observed in the female subgroup, this difference did not reach statistical significance.

Similarly, the prognostic performance of the Prognostic Nutritional Index (PNI) for DKD was evaluated (Supplementary Figure S1). In the female subgroup, the AUC for PNI was 0.705 (95%CI: 0.657–0.752). In the male subgroup, the AUC was 0.687 (95%CI: 0.651–0.724), and for the overall population, it was 0.682 (95%CI: 0.653–0.711). Consistent with the findings for CONUT, DeLong tests for PNI also revealed no statistically significant differences in AUCs when comparing the female subgroup with the male subgroup (*p* = 0.5717), the female subgroup with the overall population (*p* = 0.4312), or the male subgroup with the overall population (*p* = 0.8311). While a numerically higher AUC for PNI was observed in the female subgroup, this difference likewise did not reach statistical significance.

### 3.6 Subgroup analyses

**Figure 3:**
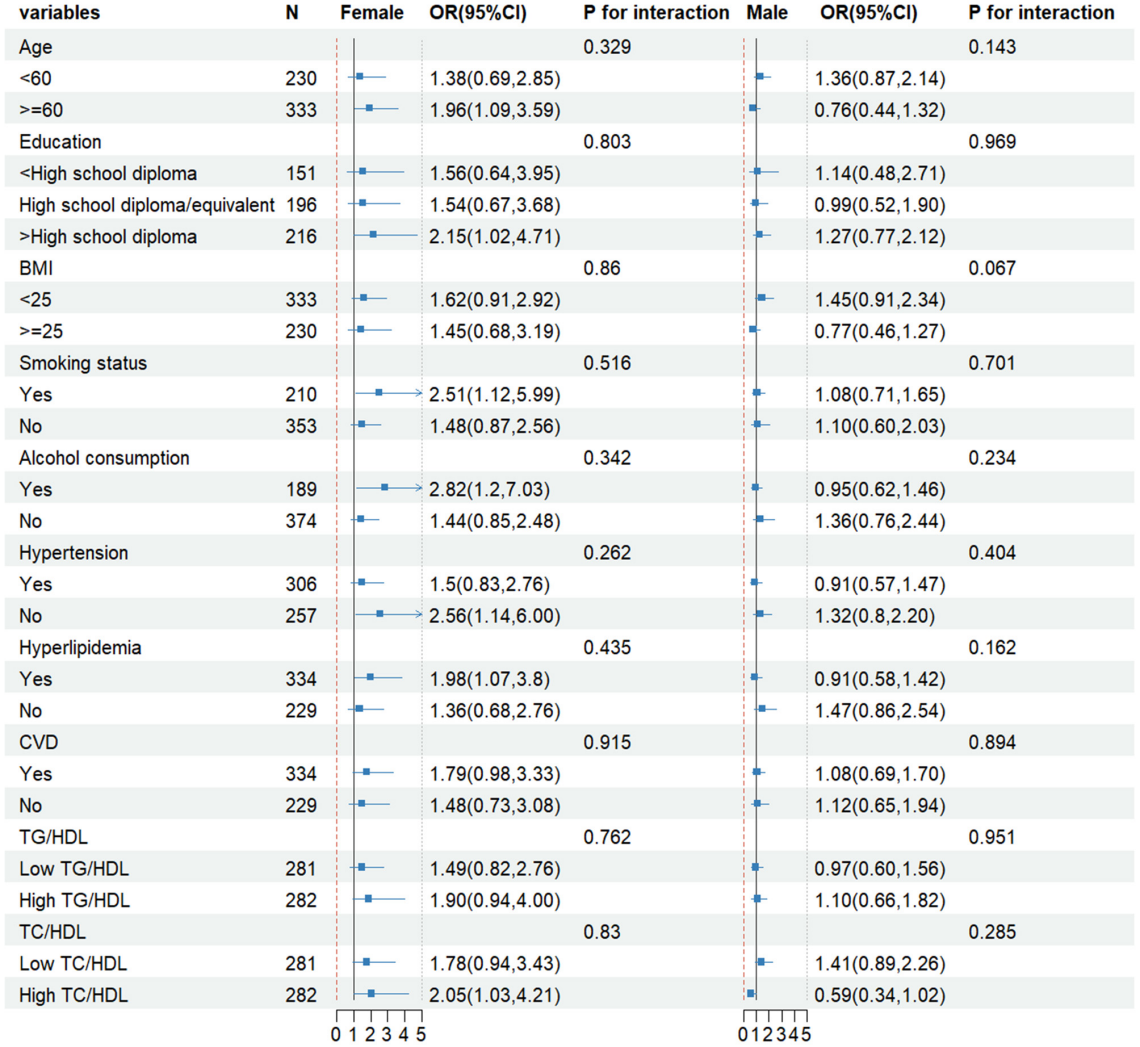
Subgroup analysis of female and male groups. The model is adjusted for age, education, marital status, BMI, hypertension, hyperlipidemia, CVD, HbA1c, DR, DPN, TF, UACR, UA, medication status, smoking, and alcohol consumption.

To determine whether a significant relationship exists between the CONUT score and DKD within specific subgroups, distinct subgroup analyses were conducted for female and male CONUT scores. Participants were classified based on age, educational attainment, BMI, marital status, smoking habits, alcohol intake, hypertension, dyslipidemia, and CVD. Subsequently, logistic regression models were applied to these subgroups. All covariates were adjusted in the models, excluding the stratification variables. As illustrated in Figure 3, a pronounced positive correlation between the CONUT score and DKD was apparent among female participants presenting with the following characteristics: age ≥ 60 years (OR = 1.96, 95%CI: 1.09–3.59), >high school diploma education (OR = 2.15, 95%CI: 1.02–4.71), smoking (OR = 2.51, 95%CI: 1.12–5.99), alcohol consumption (OR = 2.82, 95%CI: 1.20–7.03), absence of hypertension (OR = 2.56, 95%CI: 1.14–6.00), presence of dyslipidemia (OR = 1.98, 95%CI: 1.07–3.80), and those with a high TC/HDL ratio (OR = 2.05, 95%CI: 1.03–4.21). Conversely, no significant associations emerged for male participants. Furthermore, interaction *P*-values indicated no notable interplay involving the CONUT score and the various variables within either male or female sexes (P-interaction > 0.05).

### 3.7 Sensitivity analysis

To mitigate confounding effects, multivariate logistic regression analysis was additionally performed using propensity score matching (PSM). This approach accounted for diverse potential confounders inherent in the initial (unmatched) data, yielding results comparable to our primary reported estimates. As presented in Supplementary Table S5, the results from the fully adjusted model indicated that female individuals with a higher CONUT score (CONUT ≥ 3.5) demonstrated a 78% elevated odds of DKD [OR = 1.78 (95%CI: 1.07–2.99)] vs. their counterparts in the lower CONUT score category (CONUT <3.5). Conversely, no significant association between the CONUT score and DKD was detected in either the overall study population or among male individuals. The matched baseline characteristics, covariate balance plot, and probability density distribution plot can be viewed in Supplementary Table S6 and Supplementary Figures S2, S3, respectively.

## 4 Discussion

This investigation examined the association of the CONUT score with DKD prevalence within the entire study population and across different sexes. Initial analyses (Model 1 and Model 2) of the overall population indicated a notable positive correlation between the CONUT score and the odds of DKD; however, this relationship was not statistically significance in the fully adjusted model. Sex-specific analyses additionally showed an elevated CONUT score was significantly linked to heightened odds of DKD for females. In ROC curve analysis, the CONUT score demonstrated discriminatory diagnostic value in female patients. Subgroup analyses further revealed a significant positive correlation of the CONUT score with DKD among female participants who were older, possessed >high school diploma education, were smokers, did not have hypertension, or had dyslipidemia.

Previous research has established an association between nutritional risk and diabetes (26). Concomitant malnutrition in T2DM patients has been reported to elevate levels of HbA1c, RBG, insulin, and glucagon. These metabolic disturbances may be attributed to decreased insulin sensitivity and enhanced insulin resistance (27). Research by Caputo et al. (28) elucidated that, in states of malnutrition, the growth hormone/insulin-like growth factor-1 (GH/IGF-1) axis prioritizes protein preservation. This mechanism enhances lipolysis and inhibits carbohydrate oxidation. Concurrently, this axis exerts negative feedback regulation on metabolic balance, suppressing insulin signaling and promoting fatty acid oxidation, thereby affecting glycemic control and energy balance. Furthermore, beyond disrupting metabolic and endocrine functions in diabetic patients, malnutrition also profoundly modifies immune-related cytokine expression, including interleukin-2 (IL-2), interleukin-8 (IL-8), and interleukin-21 (IL-21) (29). Diminished cytokine expression compromises the immune defense capabilities of diabetic patients, rendering them more susceptible to infections. Moreover, malnutrition has been shown to exacerbate chronic inflammatory responses in diabetic patients through its influence on inflammatory mediator expression, including tumor necrosis factor-alpha (TNF-α) and interleukin-6 (IL-6) (30). Collectively, malnutrition's genesis is driven by a multifaceted interplay of metabolic dysregulation, hormonal imbalances, and immunological impairments. These processes can exacerbate systemic metabolic decline and compromise the wellbeing of individuals with diabetes by hindering nutrient absorption and assimilation. Moreover, elevated prevalence of diabetic complications and associated morbidities can further undermine nutritional status (31, 32).

Malnutrition and chronic inflammatory states exacerbate the metabolic disturbances inherent in diabetes and constitute significant risk factors for DKD onset and progression. The pathophysiology of DKD is complex, involving multiple pathways such as hyperglycemia, hemodynamic abnormalities, chronic inflammation, oxidative stress, and activation of the renin–angiotensin–aldosterone system (RAAS) (33). Malnutrition can directly or indirectly influence these pathways through various mechanisms, thereby accelerating renal damage. For instance, protein-energy wasting (PEW) is a common comorbidity in CKD patients, and its incidence is even higher in diabetic patients with co-occurring CKD (34–36). PEW is characterized by reduced body protein reserves and depleted muscle and adipose tissue, reflecting not only insufficient nutrient intake or increased nutrient loss but also close association with chronic inflammatory states (37). Hypoalbuminemia, a significant PEW marker, not only suggests inadequate protein synthesis or increased breakdown but may also exert detrimental effects on the kidneys by influencing drug binding, transport, and the regulation of vascular endothelial function. Previous research has extensively explored the association between malnutrition, inflammatory markers, and the overall DKD risk, predominantly evaluating specific nutrients, dietary patterns, or single inflammatory markers. However, research specifically addressing the sex-specific influence of nutritional status on DKD has been limited. Nevertheless, existing studies have highlighted the complex role of sex differences in DKD. For example, Clotet-Freixas et al. (38) indicated that male renal proximal tubular epithelial cells (PTECs) exhibit higher mitochondrial respiration and oxidative stress in a high-glucose environment, potentially leading to more severe cellular damage and apoptosis. In addition, male metabolic fluxes (e.g., glucose and glutamine metabolism) have been found to be significantly higher than in females under diabetic conditions, further contributing to renal damage progression. Conversely, the study by Giandalia et al. (39) suggested that while males may exhibit more prominent phenotypes of DKD (e.g., microalbuminuria), females show a higher tendency for eGFR decline and increased odds of progressing to ESRD. Notably, in post-menopausal women, the rate of renal function decline is more rapid, and females exhibit higher prevalence of non-albuminuric DKD (low eGFR type).

Beyond statistical associations, the clinical utility of the CONUT score, particularly in females, warrants specific attention. While the ROC-AUC of 0.700 for CONUT in females indicates moderate discriminatory performance for identifying individuals at risk of DKD, it is important to note that formal statistical comparisons (DeLong tests) did not show a significant difference in AUC performance when compared to males or the overall population (all *p* > 0.05). This suggests that while CONUT exhibits good discriminatory ability in females as a risk identification tool, its statistical superiority in terms of overall prediction accuracy compared to other groups was not established in this study population. This could potentially be due to a relatively small true difference in predictive ability that our sample size was underpowered to detect, or simply that the differences are not statistically meaningful. Nevertheless, our multivariate analysis did reveal a robust and significant association between elevated CONUT scores and DKD specifically in females, an association not observed in males. The CONUT score is derived from routinely available, objective, inexpensive laboratory parameters, making it an easily accessible, non-invasive, and practical tool for initial risk stratification in busy clinical settings. Its utility lies not in serving as a definitive diagnostic test alone but rather as a simple and efficient screening marker to flag high-risk female individuals who might benefit from more intensive follow-up, earlier comprehensive evaluations, or targeted nutritional interventions. In resource-limited healthcare environments or for large-scale screening, the ease of implementing the CONUT score provides a considerable advantage, offering valuable insights that can contribute to a more holistic and personalized approach to DKD prevention and management in females.

Building on prior knowledge, our investigation offers a thorough and timely evaluation of nutritional status in Chinese DKD patients, leveraging the CONUT score as a composite nutritional assessment instrument. Derived from three objective biomarkers, this score has been extensively applied in recent studies concerning diabetes and its numerous complications. Prior studies have validated its substantial clinical utility for foreseeing carotid atherosclerosis, diabetic foot ulcers, renal insufficiency, and mortality outcomes in individuals with diabetes (10, 40–42). Despite the CONUT score's established importance across diverse diabetic morbidities, studies on its connection with DKD remain limited. For example, Qu et al. (41) reported a correlation between the CONUT score and DKD, utilizing information from the National Health and Nutrition Examination Survey (NHANES) database.

Their report suggested that the CONUT score correlated positively with CKD prevalence in American T2DM patients. While that study utilized a large NHANES database, offering high sample size and representativeness, its data were sourced from the United States. The nutritional status and metabolic characteristics of Chinese T2DM patients may differ from those of the American population (43, 44), indicating that study could not fully reflect the specific circumstances of the Chinese population regarding diabetes and its complications. Furthermore, that study did not identify sex differences in the association between malnutrition and DKD. In contrast, our study, through a retrospective analysis of a Chinese population, systematically revealed sex differences in the relationship between the CONUT score and DKD for the first time, thereby expanding the applicability of existing research. Therefore, the present study's findings regarding Chinese T2DM patients address a gap existing in the literature concerning the CONUT score's association with DKD among the Chinese demographic, carrying substantial clinical implications.

When exploring sex-specific associations of the CONUT score with DKD, inherent differences in endocrine, immune, and metabolic functions between sexes warrant consideration. Our findings necessitate a deeper look into the underlying biological mechanisms, which we have extensively explored as potential hypotheses. Female physiological state is significantly influenced by sex hormones, such as estrogens and progestins, which regulate glucose and lipid metabolism, participate in immune modulation, and exert direct or indirect effects on the kidneys. Previous research suggests estrogen offers a degree of renal protection. This protective effect may be mediated by inhibiting the transforming growth factor-β(TGF-β)/Smad signaling pathway, thereby mitigating renal interstitial fibrosis. In addition, estrogen is thought to modulate the renin–angiotensin system (RAS) to reduce angiotensin II (Ang II)-mediated oxidative stress and upregulate endothelial nitric oxide synthase (eNOS) and neuronal nitric oxide synthase (nNOS) expression, which enhances nitric oxide (NO) activity and consequently improves renal hemodynamics and filtration function (45, 46). However, a significant decline in estrogen levels after menopause may accelerate CKD progression. In the context of diabetes, chronic hyperglycemia and inflammation may disrupt female sex hormone balance, leading to an increased susceptibility to metabolic disturbances and inflammation (47). Against this background, the malnutrition and chronic inflammatory state reflected by a higher CONUT score may synergistically exert negative effects with the unique female endocrine environment. This could occur particularly by exacerbating the impact of estrogen deficiency on metabolism and inflammation, or by interfering with the hormone-regulating functions of adipose tissue (48). Furthermore, chronic inflammation may directly impair sex hormone synthesis and their signaling pathways (49). This complex crosstalk among nutrition, inflammation, and endocrine factors collectively forms the pathological basis for the increased renal damage risk observed in female diabetic patients.

Notably, although the CONUT score does not directly quantify hormone levels or metabolic pathways, its constituent elements may be implicated in estrogen-related processes. ALB, a pivotal score component, functions two-fold: it indicates body protein reserves and, as a crucial transport protein, helps regulate 17β-estradiol bioavailability via low-affinity binding. This role is particularly important in maintaining free estrogen homeostasis, especially when levels of sex hormone-binding globulin (SHBG) are abnormal (50). Decreased ALB levels may affect the distribution of free estrogen, thereby influencing its interaction with estrogen receptors (ERα, ERβ) in the glomerular basement membrane and proximal tubular epithelial cells and consequently modulating associated signaling effects. Existing research indicates that abnormal expression and function of estrogen receptors (e.g., high ERβ expression in the distal tubules) are closely linked to various kidney diseases, such as vasculitis and IgA nephropathy. Furthermore, ALB itself may activate intracellular signaling pathways within renal tubular cells (e.g., NADPH oxidase and TGF-β) via oxidative stress pathways (51–53). However, the specific regulatory mechanisms of ALB on estrogen receptor function and signaling pathways require further exploration. Second, reduced peripheral lymphocyte count reflects impaired immune surveillance. Estrogen is known to promote T-cell proliferation, a Th2 bias, and regulatory T-cell (Treg) generation; thus, its deficiency or weakened signaling can lead to persistent high NF-κB-mediated pro-inflammatory cytokine expression, maintaining low-grade chronic inflammation and further accelerating glomerular filtration barrier damage (54–57). TC, another key indicator in the CONUT score, is also regulated by estrogen. Estrogen deficiency exacerbates lipid metabolic disorders, promoting glomerulosclerosis and renal tubulointerstitial fibrosis (58). Furthermore, females undergo shifts in fat distribution and metabolism across life stages, with abdominal adiposity often becoming more prominent post-menopause. Multiple studies have found a significant association between abdominal obesity (e.g., visceral fat area, waist circumference, and waist-to-hip ratio) and the risk of DKD in women (59, 60). This shift in fat distribution, by increasing metabolically active but dysfunctional visceral fat, leads to greater release of pro-inflammatory factors and adipokines. This, in turn, exacerbates systemic low-grade inflammation, insulin resistance, and dyslipidemia, directly contributing to glomerular injury and interstitial fibrosis, ultimately accelerating the progression of DKD (61).

Beyond these sex-specific physiological and metabolic considerations primarily observed in females, it is also noteworthy that our study did not find a significant association between CONUT score and DKD in males. This lack of a statistically significant association in men, despite the numerically (though not statistically significantly) lower AUC observed in males compared to females, suggests distinct underlying biological pathways or different thresholds for nutritional impact on kidney health between sexes (39, 62). Indeed, some studies indicate that males may experience more severe renal damage or a faster progression of DKD under certain conditions (63, 64). While our study design limits definitive mechanistic conclusions, this finding (i.e., the non-significant association with CONUT score in males) could imply that the CONUT score, as a composite marker, might be less sensitive to the specific pathological processes or predominant metabolic stressors leading to DKD in males. Alternatively, it might suggest that the CONUT score is more attuned to the unique metabolic and inflammatory shifts that occur in females, particularly those influenced by hormonal changes, compared to the predominant physiological processes in males leading to DKD. Further dedicated research exploring sex-specific pathophysiological differences in DKD progression and the precise utility of nutritional markers in males is warranted.

Furthermore, our subgroup analyses in females revealed a particularly strong association between elevated CONUT scores and DKD among those with >high school diploma education, smokers, alcohol consumers, those without hypertension, and those with dyslipidemia or a high TC/HDL ratio. These findings suggest that the influence of nutritional status, as assessed by the CONUT score, on DKD risk in females may be significantly modulated by specific lifestyle factors and co-morbidities. For female smokers and alcohol consumers, the heightened odds of DKD associated with elevated CONUT scores might indicate a compounding effect, where the pro-inflammatory and oxidative stress-inducing effects of smoking and alcohol synergize with the systemic inflammation and malnutrition reflected by a higher CONUT score, thereby accelerating renal damage (65–67). Similarly, in females with pre-existing dyslipidemia or a high TC/HDL ratio, an elevated CONUT score likely signifies a more profound metabolic dysregulation and systemic inflammation, which, when combined with deranged lipid profiles, can further exacerbate kidney injury (68, 69). Conversely, the stronger association observed in non-hypertensive females is particularly noteworthy; it suggests that in the absence of the well-established strong risk factor of hypertension, the nutritional and inflammatory deficits captured by the CONUT score emerge as more prominent and independent drivers of DKD (70). The findings in females with >High school diploma education also highlight specific vulnerabilities within this subgroup when nutritional status is compromised. While the exact mechanisms underlying these subgroup-specific effects warrant further investigation, these results collectively underscore the importance of comprehensive risk assessment and tailored nutritional interventions for different female patient profiles.

This study possesses several strengths. First, it represents the first systematic investigation into the sex-specific association of the CONUT score with DKD risk in a Chinese T2DM population, thereby addressing a notable gap in the existing literature concerning this specific demographic and sex-stratified analysis. Furthermore, the inclusion of the PNI for comparative analysis strengthens the study by providing a broader context for the performance of immunonutritional assessment tools in DKD. Second, these findings offer substantial clinical guidance. The results clearly elucidate the predictive value of the CONUT score for DKD risk in females, providing direct evidence-based support for implementing sex-specific nutritional risk screening and early intervention strategies in clinical practice. This, in turn, can facilitate precise management of high-risk patients. However, this study has certain limitations. First, its single-center, retrospective design might restrict the generalizability and prevent the complete exclusion of selection bias or the influence of unmeasured confounding factors on the observed associations. Second, its cross-sectional design precludes causal inference. Third, DKD diagnosis did not solely rely on renal biopsy, given its invasive nature and non-routine application. Fourth, due to the retrospective nature of our data collection and reliance on routinely available clinical parameters, we were unable to comprehensively compare the CONUT score's predictive performance with other broad-ranging nutritional assessment tools (e.g., NRS-2002 and MNA). Fifth, our discussion regarding the potential role of sex hormones (such as estrogen decline post-menopause) in mediating the observed sex-specific associations remains hypothetical as our retrospective study design did not allow for direct collection of menopausal status or circulating hormone levels. Finally, the CONUT score itself is not a specific nutritional indicator; its components can be influenced by non-nutritional factors such as coexisting inflammation, infection, or abnormal liver function. Therefore, large-scale, multi-center prospective cohort investigations are warranted to validate these conclusions and further elucidate underlying pathophysiological mechanisms, ideally incorporating data that enables comparisons with other nutritional scoring systems and comprehensive hormonal assessments.

## 5 Conclusion

An elevated CONUT score showed a significant positive correlation with DKD prevalence in females, thereby emphasizing the crucial role of nutritional status assessment in addressing diabetic complications. These results furnish valuable perspectives for advancing personalized nutritional management strategies for females with diabetes, and they establish the foundation for subsequent inquiries into nutrition-based methods focused on DKD prevention and therapy.

## Data Availability

The datasets presented in this study can be found in online repositories. The names of the repository/repositories and accession number(s) can be found below: https://osf.io/5xmqa/files/osfstorage/686cc11fdbee1267c403aac7.

## Supplementary material

Supplementary Figure S1 Dose–response relationship between PNI and overall odds of DKD and sex-specific odds of DKD among Chinese individuals.

Supplementary Figure S2 Covariate balance plot.

Supplementary Figure S3 Probability density distribution plot.

Supplementary Table S1 Calculation of CONUT score.

Supplementary Table S2 Multicollinearity diagnostics (VIF and GVIF) for variables in the fully adjusted model (Model 3) for the overall study population.

Supplementary Table S3 Multicollinearity diagnostics (VIF and GVIF) for variables in the fully adjusted model (Model 3) for female and male subgroups.

Supplementary Table S4 Association between PNI and DKD.

Supplementary Table S5 Association between CONUT score and DKD after propensity matching.

Supplementary Table S6 Baseline information from the DKD and non-DKD groups after propensity matching.
