# Supplementary figures and images for: Sex-specific associations of the controlling nutritional status score with diabetic kidney disease among Chinese individuals: a retrospective cross-sectional study
Source: Front Nutr. 2025 Sep 5;12:1662140. doi: 10.3389/fnut.2025.1662140 (PMC12447731; doi:10.3389/fnut.2025.1662140)

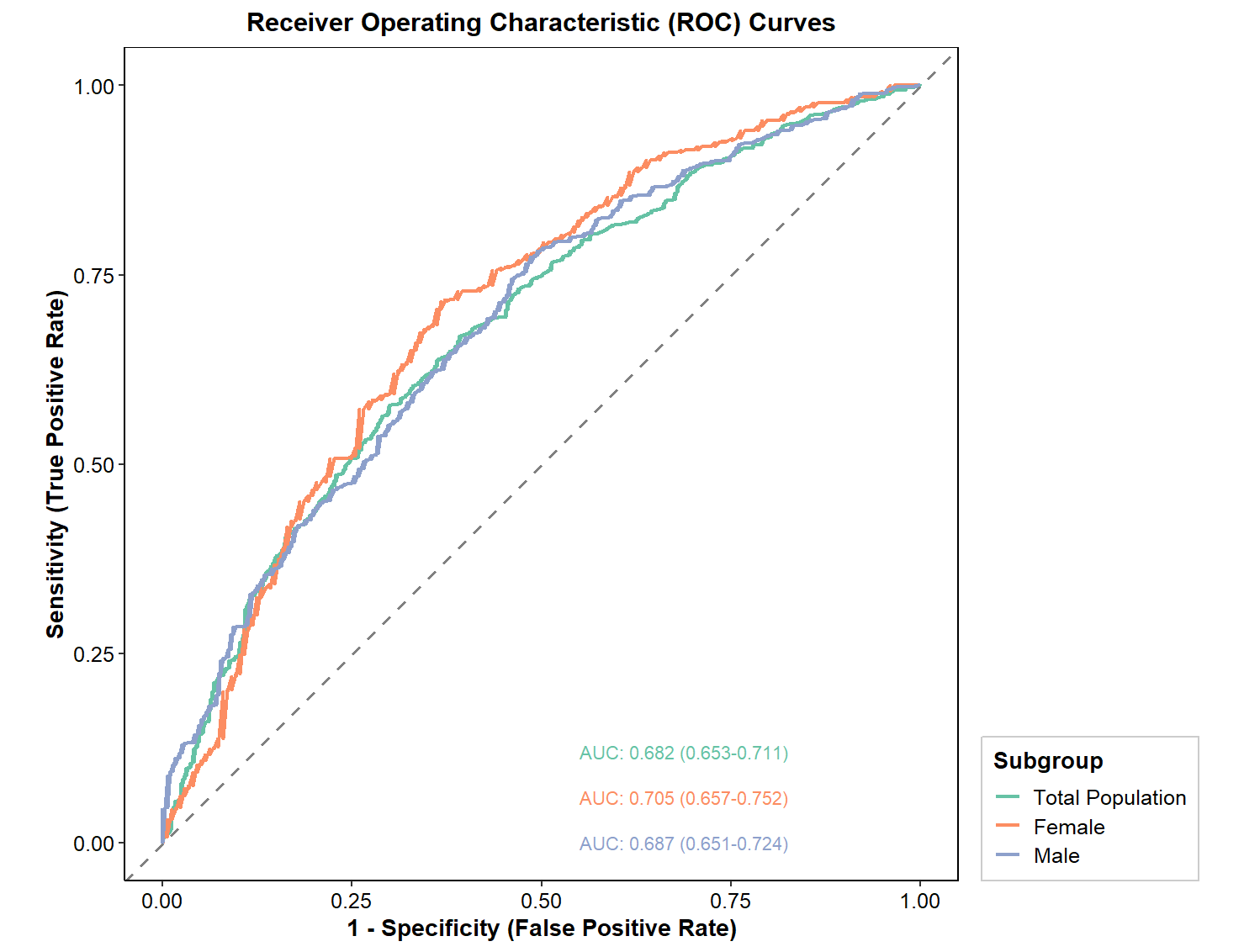

Supplement: Supplementary Figure S1 — Dose–response relationship between PNI and overall odds of DKD and sex-specific odds of DKD among Chinese individuals. [file Image_1.jpeg]

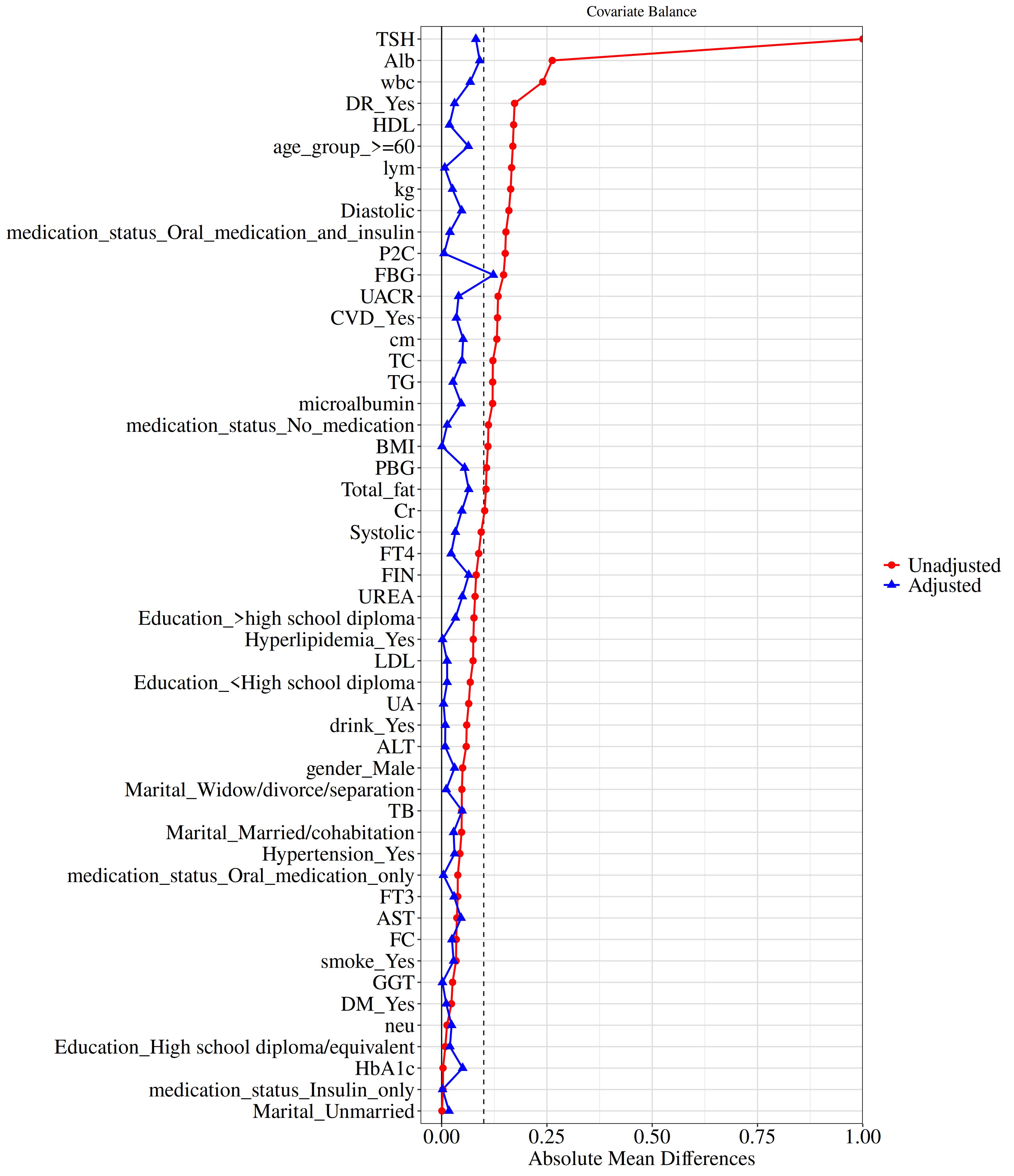

Supplement: Supplementary Figure S2 — Covariate balance plot. [file Image_2.jpeg]

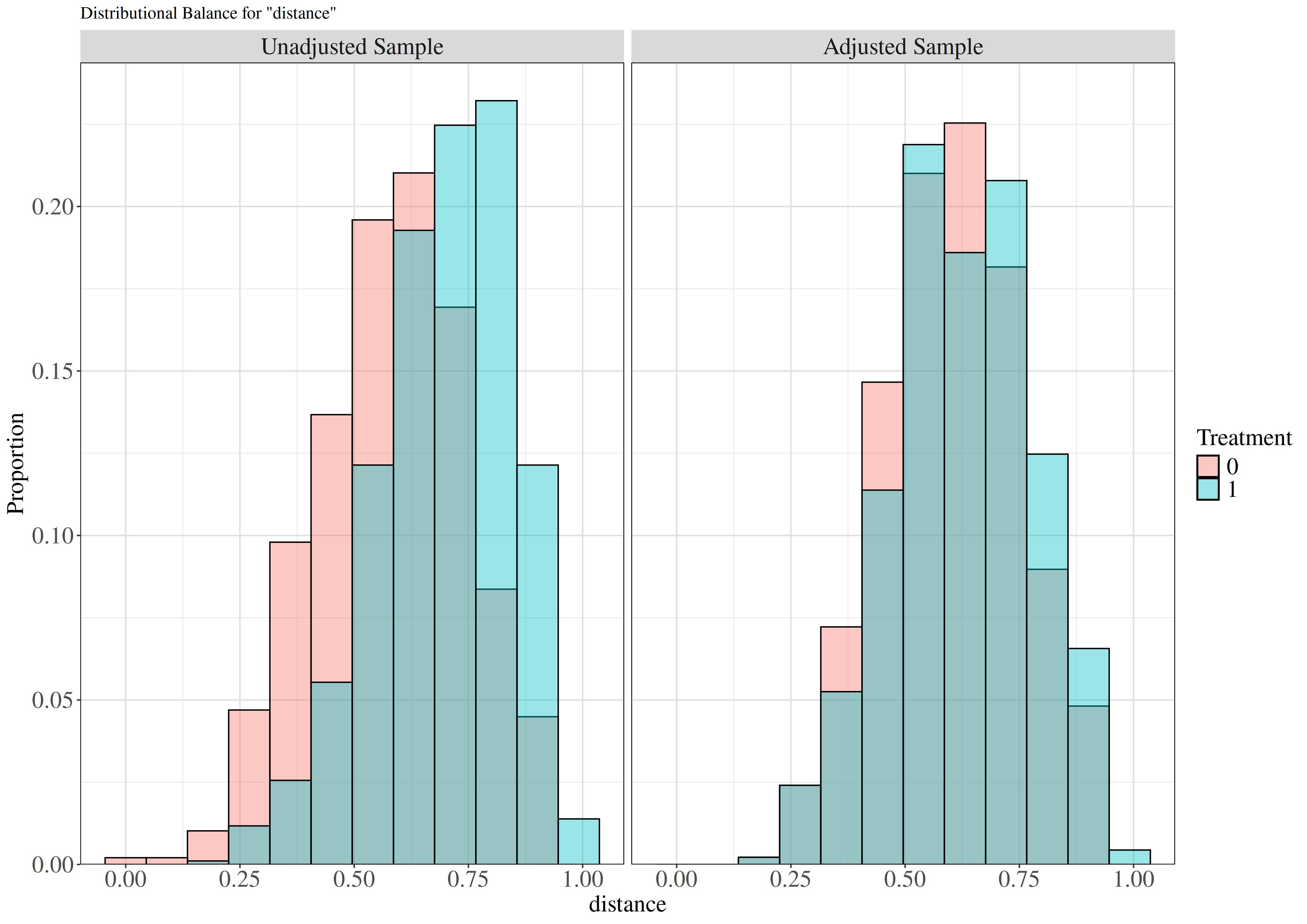

Supplement: Supplementary Figure S3 — Probability density distribution plot. [file Image_3.jpeg]
