# Supplementary material for: Sex-specific associations of the controlling nutritional status score with diabetic kidney disease among Chinese individuals: a retrospective cross-sectional study
Source: Front Nutr. 2025 Sep 5;12:1662140. doi: 10.3389/fnut.2025.1662140 (PMC12447731; doi:10.3389/fnut.2025.1662140)
Supplement: Supplementary Table S1 — Calculation of CONUT score. [file Table_1.docx]

**Supplementary Table 1.** Calculation of CONUT Score

| Parameters | CONUT | | | |
| --- | --- | --- | --- | --- |
|  | Normal | Light | Moderate | Severe |
| Serum albumin (g/dL) | 3.5-4.5 | 3.0-3.49 | 2.5-2.9 | <2.5 |
| Alb score | 1 | 2 | 4 | 6 |
| Total lymphocyte (count/mm^3^) | ≥1600 | 1200-1599 | 800-1199 | <800 |
| TLC score | 0 | 1 | 2 | 3 |
| Total cholesterol (mg/dL) | >180 | 140-180 | 100-139 | <100 |
| T-cho score | 0 | 1 | 2 | 3 |

CONUT is calculated as the sum of the Alb score, TLC score, and T-cho score

Alb albumin, TLC total lymphocytes, T-cho total cholesterol
