# Supplementary material for: Sex-specific associations of the controlling nutritional status score with diabetic kidney disease among Chinese individuals: a retrospective cross-sectional study
Source: Front Nutr. 2025 Sep 5;12:1662140. doi: 10.3389/fnut.2025.1662140 (PMC12447731; doi:10.3389/fnut.2025.1662140)
Supplement: Supplementary Table S2 — Multicollinearity diagnostics (VIF and GVIF) for variables in the fully adjusted model (Model 3) for the overall study population. [file Table_2.docx]

Supplementary Table 2: Multicollinearity Diagnostics (VIF and GVIF) for Variables in the Fully Adjusted Model (Model 3) for the Overall Study Population

| overall | | | |
| --- | --- | --- | --- |
| Variable | GVIF*^1^* | Df*^2^* | GVIF^(1/(2*Df))*^3^* |
| CONUT_group | 1.100781 | 1 | 1.04918118 |
| Gender | 1.267695 | 1 | 1.125919538 |
| Age | 1.356767 | 1 | 1.164803397 |
| Education | 1.174216 | 2 | 1.040967076 |
| Marital | 1.029942 | 2 | 1.007402812 |
| Somke | 1.311282 | 1 | 1.145112045 |
| Drink | 1.312404 | 1 | 1.145601955 |
| BMI | 1.186146 | 1 | 1.089103214 |
| Hyperlipidemia | 1.154643 | 1 | 1.074543288 |
| Hypertension | 1.192688 | 1 | 1.09210246 |
| CVD | 1.141111 | 1 | 1.06822752 |
| HbA1c | 1.092615 | 1 | 1.045282482 |
| DR | 1.053591 | 1 | 1.026445831 |
| DM | 1.208709 | 1 | 1.09941303 |
| Total fat | 1.022333 | 1 | 1.011104834 |
| UACR | 1.121983 | 1 | 1.059237188 |
| UA | 1.242915 | 1 | 1.114861058 |
| Drug | 1.134829 | 3 | 1.021304149 |

*^1^*Generalized Variance Inflation Factor, an extension of VIF, used particularly for assessing multicollinearity when categorical variables are represented by multiple dummy variables in the model.

*^2^*Degrees of freedom associated with the variable.

*^3^*The recommended diagnostic for assessing multicollinearity, especially for variables with Df > 1. For variables with Df = 1, GVIF is equivalent to VIF, and GVIF^(1/(2Df)) is its square root. Values of GVIF^(1/(2Df)) less than 2 (or VIF less than 10 for Df=1) are generally considered to indicate no problematic multicollinearity. In this table, all values are below these thresholds, suggesting no significant multicollinearity.
