# Supplementary material for: Sex-specific associations of the controlling nutritional status score with diabetic kidney disease among Chinese individuals: a retrospective cross-sectional study
Source: Front Nutr. 2025 Sep 5;12:1662140. doi: 10.3389/fnut.2025.1662140 (PMC12447731; doi:10.3389/fnut.2025.1662140)
Supplement: Supplementary Table S3 — Multicollinearity diagnostics (VIF and GVIF) for variables in the fully adjusted model (Model 3) for female and male subgroups. [file Table_3.docx]

Supplementary Table 3: Multicollinearity Diagnostics (VIF and GVIF) for Variables in the Fully Adjusted Model (Model 3) for Female and Male Subgroups

| female | | | | male | | |
| --- | --- | --- | --- | --- | --- | --- |
| Variable | GVIF*^1^* | Df*^2^* | GVIF^(1/(2*Df))*^3^* | GVIF*^1^* | Df*^2^* | GVIF^(1/(2*Df))*^3^* |
| CONUT_group | 1.097933 | 1 | 1.047822908 | 1.134426 | 1 | 1.065094219 |
| Age | 1.315612 | 1 | 1.147001227 | 1.336987 | 1 | 1.156281737 |
| Education | 1.310334 | 2 | 1.06990579 | 1.144398 | 2 | 1.034294548 |
| Marital | 1.063199 | 2 | 1.015438646 | 1.044362 | 2 | 1.010910615 |
| Somke | 1.514895 | 1 | 1.230810715 | 1.167224 | 1 | 1.080381402 |
| Drink | 1.500141 | 1 | 1.224802545 | 1.151327 | 1 | 1.07299928 |
| BMI | 1.152181 | 1 | 1.073396964 | 1.226953 | 1 | 1.107679102 |
| Hyperlipidemia | 1.233385 | 1 | 1.110578739 | 1.157651 | 1 | 1.075941949 |
| Hypertension | 1.269556 | 1 | 1.126745692 | 1.177046 | 1 | 1.084917364 |
| CVD | 1.196089 | 1 | 1.093658676 | 1.143271 | 1 | 1.069238573 |
| HbA1c | 1.113121 | 1 | 1.055045508 | 1.128654 | 1 | 1.062381238 |
| DR | 1.11745 | 1 | 1.057095156 | 1.047992 | 1 | 1.023714578 |
| DM | 1.223993 | 1 | 1.106342315 | 1.222495 | 1 | 1.105665079 |
| Total fat | 1.07441 | 1 | 1.036537718 | 1.023882 | 1 | 1.011870536 |
| UACR | 1.141033 | 1 | 1.068191342 | 1.125596 | 1 | 1.060941306 |
| UA | 1.120135 | 1 | 1.058364155 | 1.164014 | 1 | 1.078894736 |
| Drug | 1.285564 | 3 | 1.042754982 | 1.137602 | 3 | 1.02171961 |

*^1^*Generalized Variance Inflation Factor, an extension of VIF, used particularly for assessing multicollinearity when categorical variables are represented by multiple dummy variables in the model.

*^2^*Degrees of freedom associated with the variable.

*^3^*The recommended diagnostic for assessing multicollinearity, especially for variables with Df > 1. For variables with Df = 1, GVIF is equivalent to VIF, and GVIF^(1/(2Df)) is its square root. Values of GVIF^(1/(2Df)) less than 2 (or VIF less than 10 for Df=1) are generally considered to indicate no problematic multicollinearity. In this table, all values are below these thresholds, suggesting no significant multicollinearity.
