# Supplementary material for: Sex-specific associations of the controlling nutritional status score with diabetic kidney disease among Chinese individuals: a retrospective cross-sectional study
Source: Front Nutr. 2025 Sep 5;12:1662140. doi: 10.3389/fnut.2025.1662140 (PMC12447731; doi:10.3389/fnut.2025.1662140)
Supplement: Supplementary Table S4 — Association between PNI and DKD. [file Table_4.docx]

**Table S4.** **The Association Between PNI and Diabetic Kidney Disease (DKD).**

| **Variable** | **Characteristic** | **Model1 OR (95%CI)** | **P-value** | **Model2 OR (95%CI)** | **P-value** | **Model3 OR (95%CI)** | **P-value** |
| --- | --- | --- | --- | --- | --- | --- | --- |
| Overall | PNI |  |  |  |  |  |  |
|  | <50.55 | ref |  | ref |  | ref |  |
|  | ≥50.55 | 0.60(0.48,0.75) | <0.001 | 0.69(0.55,0.87) | 0.002 | 0.74(0.58,0.95) | 0.018 |
| Female | PNI |  |  |  |  |  |  |
|  | <50.55 | ref |  | ref |  | ref |  |
|  | ≥50.55 | 0.65(0.45.0.94) | 0.021 | 0.70(0.48,1.02) | 0.062 | 0.70(0.46,1.06) | 0.090 |
| Male | PNI |  |  |  |  |  |  |
|  | <50.55 | ref |  | ref |  | ref |  |
|  | ≥50.55 | 0.59(0.44,0.77) | <0.001 | 0.69(0.51,0.92) | 0.013 | 1.07(0.77,1.49) | 0.057 |

**Notes:** Data are presented as weighted odds ratios (OR) with 95% confidence intervals (CI). Model 1 is the crude model. Model 2 is adjusted for gender, age, education, marital status. Model 3 is further adjusted for BMI, hypertension, hyperlipidemia, CVD, HbA1c, DR, DPN, TF, UACR, UA, medication status, smoking, and alcohol consumption.
