# Supplementary material for: Sex-specific associations of the controlling nutritional status score with diabetic kidney disease among Chinese individuals: a retrospective cross-sectional study
Source: Front Nutr. 2025 Sep 5;12:1662140. doi: 10.3389/fnut.2025.1662140 (PMC12447731; doi:10.3389/fnut.2025.1662140)
Supplement: Supplementary Table S5 — Association between CONUT score and DKD after propensity matching. [file Table_5.docx]

**Table S5.** **The Association Between CONUT Score and Diabetic Kidney Disease (DKD) After Propensity Matching.**

| **Variable** | **Characteristic** | **Model1 OR (95%CI)** | **P-value** | **Model2 OR (95%CI)** | **P-value** | **Model3 OR (95%CI)** | **P-value** |
| --- | --- | --- | --- | --- | --- | --- | --- |
| Overall | CONUT |  |  |  |  |  |  |
|  | <3.5 | ref |  | ref |  | ref |  |
|  | ≥3.5 | 1.20(0.91,1.59) | 0.2 | 1.15(0.87,1.53) | 0.3 | 1.12(0.84,1.50) | 0.4 |
| Female | CONUT |  |  |  |  |  |  |
|  | <3.5 | ref |  | ref |  | ref |  |
|  | ≥3.5 | 1.56(0.99,2.46) | 0.055 | 1.55(0.98,2.48) | 0.063 | 1.78(1.07,2.99) | 0.028 |
| Male | CONUT |  |  |  |  |  |  |
|  | <3.5 | ref |  | ref |  | ref |  |
|  | ≥3.5 | 1.03(0.73,1.46) | 0.9 | 0.96(0.67,1.38) | 0.8 | 1.94(0.65,1.38) | 0.8 |

**Notes:** Data are presented as weighted odds ratios (OR) with 95% confidence intervals (CI). Model 1 is the crude model. Model 2 is adjusted for gender, age, education, marital status. Model 3 is further adjusted for BMI, hypertension, hyperlipidemia, CVD, HbA1c, DR, DPN, TF, UACR, UA, medication status, smoking, and alcohol consumption.
