# Supplementary material for: Sex-specific associations of the controlling nutritional status score with diabetic kidney disease among Chinese individuals: a retrospective cross-sectional study
Source: Front Nutr. 2025 Sep 5;12:1662140. doi: 10.3389/fnut.2025.1662140 (PMC12447731; doi:10.3389/fnut.2025.1662140)
Supplement: Supplementary Table S6 — Baseline information from the DKD and non-DKD groups after propensity matching. [file Table_6.docx]

**Table S6. Baseline information from the DKD and Non-DKD groups after propensity matching.**

| Variable | Total  (n = 914) | Non-DKD  (n = 457) | DKD  (n = 457) | Statistic | *P* | SMD |
| --- | --- | --- | --- | --- | --- | --- |
|  |  |  |  |  |  |  |
| Cm, M (Q₁, Q₃) | 1.66 (1.60, 1.70) | 1.66 (1.60, 1.70) | 1.65 (1.60, 1.70) | Z=-0.590 | 0.555 | -0.052 |
| Kg, M (Q₁, Q₃) | 68.00 (60.00, 75.75) | 68.00 (60.00, 76.00) | 68.00 (60.00, 75.00) | Z=-0.143 | 0.886 | -0.027 |
| Diastolic, M (Q₁, Q₃) | 82.00 (75.00, 89.00) | 82.00 (76.00, 90.00) | 82.00 (74.00, 89.00) | Z=-1.370 | 0.171 | -0.042 |
| Systolic, M (Q₁, Q₃) | 131.00 (119.00, 145.00) | 131.00 (119.00, 145.00) | 131.00 (119.00, 145.00) | Z=-0.268 | 0.788 | 0.034 |
| Wbc, M (Q₁, Q₃) | 5.89 (4.92, 7.09) | 5.89 (5.00, 7.10) | 5.89 (4.88, 7.08) | Z=-0.694 | 0.488 | -0.067 |
| Neu, M (Q₁, Q₃) | 3.38 (2.72, 4.22) | 3.41 (2.68, 4.19) | 3.32 (2.75, 4.23) | Z=-0.604 | 0.546 | -0.065 |
| Lym, M (Q₁, Q₃) | 1.81 (1.44, 2.20) | 1.80 (1.44, 2.20) | 1.82 (1.44, 2.20) | Z=-0.059 | 0.953 | -0.010 |
| Alb, M (Q₁, Q₃) | 4.12 (3.92, 4.36) | 4.14 (3.94, 4.36) | 4.10 (3.90, 4.35) | Z=-1.494 | 0.135 | -0.089 |
| TB, M (Q₁, Q₃) | 12.80 (9.90, 16.20) | 12.90 (10.10, 16.60) | 12.50 (9.60, 15.90) | Z=-1.185 | 0.236 | -0.052 |
| UREA, M (Q₁, Q₃) | 5.85 (4.89, 6.97) | 5.76 (4.83, 6.87) | 5.94 (4.91, 7.11) | Z=-1.080 | 0.280 | 0.040 |
| Cr, M (Q₁, Q₃) | 68.05 (55.92, 81.50) | 67.30 (56.60, 81.10) | 69.00 (55.30, 82.20) | Z=-0.466 | 0.641 | 0.048 |
| UA, M (Q₁, Q₃) | 314.25 (262.45, 377.05) | 316.60 (266.80, 377.50) | 310.60 (258.80, 376.50) | Z=-0.547 | 0.584 | 0.004 |
| ALT, M (Q₁, Q₃) | 14.00 (10.00, 22.00) | 14.00 (10.00, 23.00) | 14.00 (9.00, 21.00) | Z=-1.365 | 0.172 | -0.007 |
| AST, M (Q₁, Q₃) | 18.00 (14.00, 22.00) | 17.00 (14.00, 22.00) | 18.00 (14.00, 22.00) | Z=-0.049 | 0.961 | 0.039 |
| GGT, M (Q₁, Q₃) | 25.00 (17.00, 37.00) | 25.00 (17.00, 38.00) | 23.00 (16.00, 36.00) | Z=-1.670 | 0.095 | -0.004 |
| Microalbumin, M (Q₁, Q₃) | 8.56 (5.17, 25.36) | 8.41 (5.20, 24.44) | 8.82 (5.05, 25.78) | Z=-0.285 | 0.776 | 0.053 |
| UACR, M (Q₁, Q₃) | 8.36 (4.97, 21.80) | 7.74 (4.83, 20.09) | 9.13 (5.12, 23.66) | Z=-1.984 | 0.047 | 0.057 |
| HbA1c, M (Q₁, Q₃) | 8.30 (7.10, 9.90) | 8.30 (7.10, 9.80) | 8.30 (7.10, 10.00) | Z=-0.278 | 0.781 | 0.048 |
| FBG, M (Q₁, Q₃) | 7.92 (6.48, 9.63) | 7.87 (6.48, 9.44) | 8.00 (6.48, 9.94) | Z=-1.276 | 0.202 | 0.125 |
| PBG, M (Q₁, Q₃) | 13.54 (11.07, 16.94) | 13.70 (10.89, 16.81) | 13.35 (11.21, 17.12) | Z=-0.539 | 0.590 | 0.056 |
| FIN, M (Q₁, Q₃) | 7.59 (4.88, 12.03) | 7.50 (4.86, 11.52) | 7.64 (4.97, 12.29) | Z=-0.525 | 0.600 | 0.064 |
| P2C, M (Q₁, Q₃) | 3.38 (2.29, 4.70) | 3.39 (2.30, 4.66) | 3.37 (2.25, 4.78) | Z=-0.236 | 0.813 | 0.005 |
| Total fat, M (Q₁, Q₃) | 63.78 (42.69, 94.85) | 64.31 (42.45, 98.27) | 63.09 (43.04, 91.23) | Z=-0.701 | 0.483 | -0.062 |
| FC, M (Q₁, Q₃) | 1.59 (1.13, 2.17) | 1.59 (1.12, 2.16) | 1.59 (1.14, 2.18) | Z=-0.348 | 0.728 | 0.026 |
| TSH, M (Q₁, Q₃) | 1.74 (1.21, 2.48) | 1.71 (1.18, 2.45) | 1.77 (1.21, 2.51) | Z=-1.210 | 0.226 | 0.111 |
| FT3, M (Q₁, Q₃) | 2.68 (2.44, 2.91) | 2.70 (2.47, 2.93) | 2.66 (2.42, 2.89) | Z=-1.427 | 0.154 | 0.027 |
| FT4, M (Q₁, Q₃) | 13.75 (12.68, 14.81) | 13.73 (12.69, 14.89) | 13.76 (12.67, 14.70) | Z=-0.301 | 0.763 | -0.021 |
| HDL, M (Q₁, Q₃) | 1.04 (0.89, 1.24) | 1.04 (0.89, 1.24) | 1.03 (0.89, 1.23) | Z=-0.096 | 0.924 | -0.020 |
| LDL, M (Q₁, Q₃) | 2.34 (1.80, 2.82) | 2.34 (1.82, 2.81) | 2.35 (1.78, 2.83) | Z=-0.265 | 0.791 | -0.014 |
| TG, M (Q₁, Q₃) | 1.69 (1.19, 2.50) | 1.72 (1.21, 2.53) | 1.67 (1.18, 2.49) | Z=-0.764 | 0.445 | -0.020 |
| TC, M (Q₁, Q₃) | 80.89 (68.46, 94.00) | 81.43 (69.18, 93.50) | 80.17 (67.92, 94.04) | Z=-0.920 | 0.358 | -0.047 |
| BMI, M (Q₁, Q₃) | 24.80 (22.84, 27.09) | 24.79 (22.86, 27.06) | 24.97 (22.83, 27.10) | Z=-0.226 | 0.821 | 0.001 |
| Gender, n (%) |  |  |  | χ²=0.916 | 0.339 |  |
| Female | 342 (37.42) | 164 (35.89) | 178 (38.95) |  |  | 0.063 |
| Male | 572 (62.58) | 293 (64.11) | 279 (61.05) |  |  | -0.063 |
| Education, n (%) |  |  |  | χ²=0.991 | 0.609 |  |
| <High school diploma | 188 (20.57) | 91 (19.91) | 97 (21.23) |  |  | 0.032 |
| >high school diploma | 423 (46.28) | 219 (47.92) | 204 (44.64) |  |  | -0.066 |
| High school diploma/equivalent | 303 (33.15) | 147 (32.17) | 156 (34.14) |  |  | 0.042 |
| Marital, n (%) |  |  |  | χ²=1.767 | 0.413 |  |
| Married/cohabitation | 597 (65.32) | 292 (63.89) | 305 (66.74) |  |  | 0.060 |
| Unmarried | 46 (5.03) | 27 (5.91) | 19 (4.16) |  |  | -0.088 |
| Widow/divorce/separation | 271 (29.65) | 138 (30.20) | 133 (29.10) |  |  | -0.024 |
| DR, n (%) |  |  |  | χ²=1.030 | 0.310 |  |
| No | 644 (70.46) | 329 (71.99) | 315 (68.93) |  |  | -0.066 |
| Yes | 270 (29.54) | 128 (28.01) | 142 (31.07) |  |  | 0.066 |
| DM, n (%) |  |  |  | χ²=0.119 | 0.730 |  |
| No | 589 (64.44) | 297 (64.99) | 292 (63.89) |  |  | -0.023 |
| Yes | 325 (35.56) | 160 (35.01) | 165 (36.11) |  |  | 0.023 |
| Hypertension, n (%) |  |  |  | χ²=0.859 | 0.354 |  |
| No | 440 (48.14) | 227 (49.67) | 213 (46.61) |  |  | -0.061 |
| Yes | 474 (51.86) | 230 (50.33) | 244 (53.39) |  |  | 0.061 |
| Hyperlipidemia, n (%) |  |  |  | χ²=0.005 | 0.946 |  |
| No | 343 (37.53) | 171 (37.42) | 172 (37.64) |  |  | 0.005 |
| Yes | 571 (62.47) | 286 (62.58) | 285 (62.36) |  |  | -0.005 |
| CVD, n (%) |  |  |  | χ²=1.127 | 0.288 |  |
| No | 422 (46.17) | 219 (47.92) | 203 (44.42) |  |  | -0.070 |
| Yes | 492 (53.83) | 238 (52.08) | 254 (55.58) |  |  | 0.070 |
| Smoke, n (%) |  |  |  | χ²=0.747 | 0.387 |  |
| No | 411 (44.97) | 199 (43.54) | 212 (46.39) |  |  | 0.057 |
| Yes | 503 (55.03) | 258 (56.46) | 245 (53.61) |  |  | -0.057 |
| Drink, n (%) |  |  |  | χ²=0.070 | 0.791 |  |
| No | 428 (46.83) | 212 (46.39) | 216 (47.26) |  |  | 0.018 |
| Yes | 486 (53.17) | 245 (53.61) | 241 (52.74) |  |  | -0.018 |
| Medication Status, n (%) |  |  |  | χ²=0.515 | 0.916 |  |
| Insulin_only | 25 (2.74) | 13 (2.84) | 12 (2.63) |  |  | -0.014 |
| No_medication | 146 (15.97) | 76 (16.63) | 70 (15.32) |  |  | -0.036 |
| Oral_medication_and_insulin | 373 (40.81) | 182 (39.82) | 191 (41.79) |  |  | 0.040 |
| Oral_medication_only | 370 (40.48) | 186 (40.70) | 184 (40.26) |  |  | -0.009 |
| Age Group, n (%) |  |  |  | χ²=3.836 | 0.050 |  |
| <60 | 549 (60.07) | 289 (63.24) | 260 (56.89) |  |  | -0.128 |
| >=60 | 365 (39.93) | 168 (36.76) | 197 (43.11) |  |  | 0.128 |

**Z:** Mann-Whitney test, χ²: Chi-square test

**M:** Median, Q₁: 1st Quartile, Q₃: 3st Quartile

**Abbreviations:** BMI, Body mass index; DBP, Diastolic blood pressure; SBP, Systolic blood pressure; WBC, White blood cell; NEU, Neutrophil;

LYM, Lymphocyte; ALB, Albumin; TB, Total bilirubin; Cr, Creatinine; UA, Uric acid; ALT, Alanine aminotransferase; AST, Aspartate aminotransferase; GGT, Gamma-glutamyl transferase; µALB, Microalbumin; UACR, Urinary albumin-to-creatinine ratio; FBG, Fasting blood glucose; PBG, Postprandial blood glucose; FI, Fasting insulin; 2h-CP, Postprandial C-peptide; TF, Total fat; FCP, Fasting C-peptide; TSH, Thyroid stimulating hormone; FT3, Free Triiodothyronine; FT4, Free Thyroxine; HDL, High density lipoprotein; LDL, Low density lipoprotein; TG, Triglycerides; TC, Total cholesterol.
